# Supplementary material for: Complexome profiling of the Chlamydomonas psb28 mutant reveals TEF5 as an early PSII assembly factor
Source: Plant Cell. 2025 Mar 17;37(6):koaf055. doi: 10.1093/plcell/koaf055 (PMC12164590; doi:10.1093/plcell/koaf055)
Supplement: koaf055_Supplementary_Data [file koaf055_supplementary_data.zip › psb28_interactive.zip!/plots/intensity/qualityPlots/peptides/_Cre06.g279850.t1.2.html]

### Cre06.g279850.t1.2

Trivial name:   
SeqCoverage [median]: 17.70  
Mass: 28.97 kDa  
  
 

| Sequence | MissedCleavage | Mass [Da] | wtIntensity | muIntensity | Score |
| --- | --- | --- | --- | --- | --- |
| AQIDAAK | 0 | 715.39 | 292.05 | 0.00 | 84.24 |
| KVVLVSSMGGTDPSNNLNK | 1 | 1959.01 | 0.00 | 566.16 | 45.14 |
| LGVDDTLIKETVR | 1 | 1457.81 | 633.84 | 1317.66 | 34.44 |
| VAVTGAAGR | 0 | 800.45 | 0.00 | 462.01 | 91.85 |
|  |  |  | 925.9 | 2345.8 |  |
